# Measurements of continuum lowering in solid-density plasmas created from elements and compounds

**DOI:** 10.1038/ncomms11713

**Published:** 2016-05-23

**Authors:** O. Ciricosta, S. M. Vinko, B. Barbrel, D. S. Rackstraw, T. R. Preston, T. Burian, J. Chalupský, B. I. Cho, H. -K. Chung, G. L. Dakovski, K. Engelhorn, V. Hájková, P. Heimann, M. Holmes, L. Juha, J. Krzywinski, R. W. Lee, S. Toleikis, J. J. Turner, U. Zastrau, J. S. Wark

**Affiliations:** 1 Clarendon Laboratory, Department of Physics, University of Oxford, Parks Road, Oxford OX1 3PU, UK; 2 Physics Department, UC Berkeley, LeConte Hall, Berkeley, California 94720, USA; 3 Institute of Physics ASCR, Na Slovance 2, 18221 Prague 8, Czech Republic; 4 Center for Relativistic Laser Science, Institute for Basic Science (IBS), Gwangju 500-712, Korea; 5 Department of Physics and Photon Science, Gwangju Institute of Science and Technology, Gwangju 500-712, Korea; 6 Atomic and Molecular Data Unit, Nuclear Data Section, IAEA, P.O. Box 100, Vienna A-1400, Austria; 7 SLAC National Accelerator Laboratory, 2575 Sand Hill Road, Menlo Park, California 94025, USA; 8 Lawrence Berkeley National Laboratory, 1 Cyclotron Road, Berkeley, California 94720, USA; 9 Department of Physics, University of California, Berkeley, California 94720, USA; 10 Deutsches-Elektronensynchrotron DESY, Notkestrasse 85, 22603 Hamburg, Germany; 11 IOQ, Friedrich-Schiller-Universität Jena, Max-Wien-Platz 1, 07743 Jena, Germany

## Abstract

The effect of a dense plasma environment on the energy levels of an embedded ion is usually described in terms of the lowering of its continuum level. For strongly coupled plasmas, the phenomenon is intimately related to the equation of state; hence, an accurate treatment is crucial for most astrophysical and inertial-fusion applications, where the case of plasma mixtures is of particular interest. Here we present an experiment showing that the standard density-dependent analytical models are inadequate to describe solid-density plasmas at the temperatures studied, where the reduction of the binding energies for a given species is unaffected by the different plasma environment (ion density) in either the element or compounds of that species, and can be accurately estimated by calculations only involving the energy levels of an isolated neutral atom. The results have implications for the standard approaches to the equation of state calculations.

## Abstract (web summary)

The effect of dense plasma environment on the energy levels of an ion is usually described in terms of a lowering of its continuum level. Here the authors present an isochoric-heating experiment to measure and compare continuum lowering in single-species and mixture plasmas to provide insights for models.

## Graphical abstract

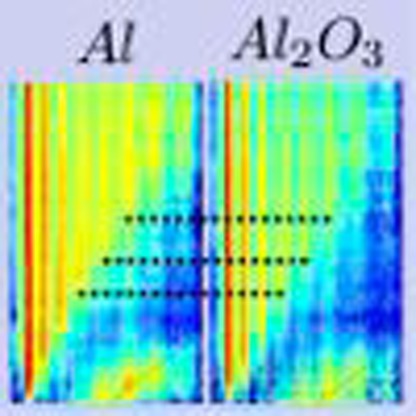

A correct description of how atoms and ions interact with each other in a dense system is of fundamental importance across a wide range of disciplines: in the case of a plasma, the effect of the charged environment on a single ion is usually represented in terms of a lowering of its continuum level^1^,^2^. This energy shift results in a modification of the ionization balance in the plasma, directly related to important properties of the system such as its opacity and equation of state (EOS)^3^, which play a pivotal role in our understanding of solar^4^, planetary^5^ and inertial-fusion^6^ systems.

For strongly coupled plasmas (high density and moderate temperature), the plasma density is a key parameter in any continuum lowering or EOS model, either by being a quantity explicitly included in analytical approaches^1^,^2^,^3^ or by determining the boundary conditions in non-analytical calculations^7^,^8^,^9^,^10^. Unfortunately, the inevitable density gradients in standard laser-plasma experiments and the lack of benchmarked density diagnostics for high-density plasmas have both hindered a rigorous experimental validation of these widely used models. Only with the recent development of X-ray free-electron lasers (FELs) has a new class of experiments become available. An 80-fs X-ray pulse has been proven to create an ∼200-eV plasma from an Al foil, detectable through a *K*_α_ fluorescence signal limited to the same timescale, during which no plasma expansion can occur^11^: this isochoric heating experiment has allowed for the first charge-resolved measurement of continuum lowering in a hot-dense plasma at a precisely known density^12^, which has shown that the most widely used model^2^ can substantially underestimate this effect, renewing the interest of the plasma community^10^,^11^,^12^,^13^,^14^,^15^,^16^,^17^ in the problem.

In the above context we present the results of an FEL isochoric-heating experiment, where we have measured the continuum lowering in both single-species and mixture plasmas, and the comparison between measurements on the same ion species in different materials allows for a measurement at different—yet still precisely known—densities. The experiment also provides insight on the treatment of mixtures, of particular interest in solar and inertial-fusion science, where the properties of dopant species are essential for a complete understanding of the relevant systems^4^,^18^,^19^,^20^. Our results imply that a correct interpretation of continuum-lowering effects cannot be based only on the average plasma density (defined as the average ionization times the ion density) and suggest that orbital overlap effects between neighbouring ions need to be included in the numerical models.

## Results

### *K*-edge measurements

We have used the focused X-ray beam of the Linac Coherent Light Source (LCLS)^21^ to isochorically heat thin foils of Mg, Al, Si, Al_2_O_3_, SiO_2_ and mica (KAl_3_Si_3_O_12_H_2_) to the hot-dense regime. Pulses of X-rays with a nominal duration of 100 fs were focused to intensities exceeding 10^17^ W cm^−2^ and the laser photon energy was varied above the cold *K*-edge of Mg, Al or Si, in the range between 1,290 and 1,985 eV. It has been previously shown for aluminium^11^ that in these conditions the sample is isochorically heated to electron temperatures up to 200 eV and the main laser absorption mechanism—the photoionization of *K*-shell electrons—also provides an excellent plasma diagnostic, owing to the subsequent radiative recombination of *L*-shell electrons into the *K*-shell holes (*K*_α_ spectroscopy). In particular, by observing the onset of the *K*_α_ fluorescence from ions with a different number of *L*-shell holes (*K*_α_ satellites) as a function of the laser photon energy, a charge-resolved measurement of the *K*-edges, and thus of continuum lowering^12^, can be performed in a system where the ion density is exactly known, being equal to the solid density value.

**Figure 1:**
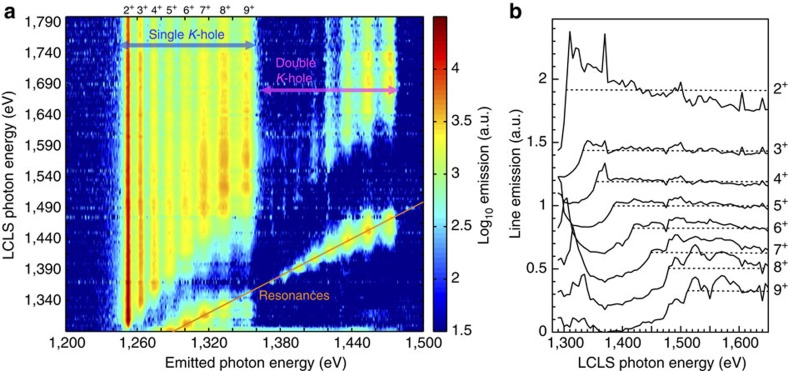
Recorded *K*_α_ emission in magnesium. The emission spectral intensity for a Mg target pumped by the LCLS is plotted in **a** on a logarithmic colour scale, as a function of the emitted and pumping photon energy. The two observed series correspond to *K*_α_ emission lines from ions with either one or two holes in the *K*-shell (generated by photoabsorption), where each line within a series arises from ions with a given number of electrons in the *L*-shell. The charge states labelling the lines of the main sequence are those of the corresponding absorbing ions, before the *K*-shell photoionization process has occurred; 2^+^ is the ground state of the Mg ions in the initial metallic state. The orange line corresponds to the condition *x*=*y*, where the hole in the *K*-shell for the initial state of the *K*_α_ transition is generated by resonant pumping of a *K*-shell electron to the *L*-shell. In **b**, vertical lineouts for the emission intensity from each of the labelled charge states in **a** is plotted as a function of the LCLS photon energy. The curves have been shifted vertically for clarity. The dotted lines are meant to guide the eye for the identification of the plateau, which corresponds to LCLS photon energies in excess of the *K*-edge threshold for the given ion. The pre-edge peaks at low energies correspond to the resonances. Features that are common between different charge states are due either to collisional effects (see text) or to the varying signal-to-noise ratio with the pump photon energy (see Methods section).

**Figure 2:**
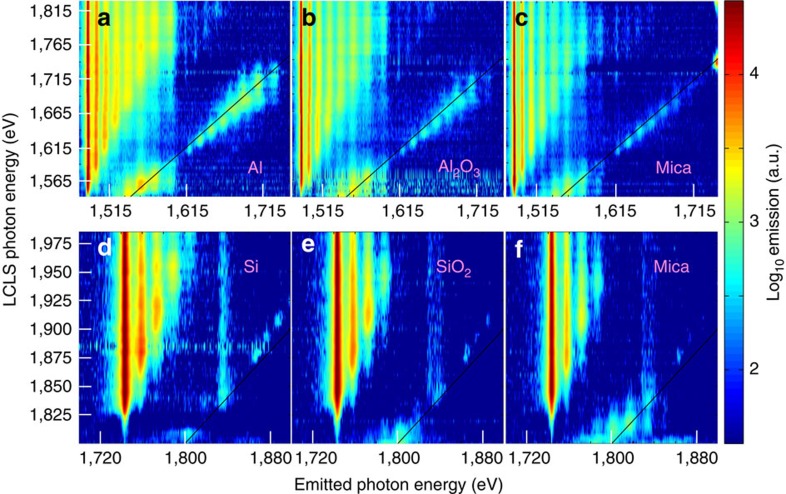
Emission spectra for Al and Si in different materials. The Al *K*_α_ spectra observed when the LCLS pumps either a pure aluminium (**a**), an alumina (**b**) or a mica sample (**c**) are plotted as a function of the emitted and pumping photon energy, on a logarithmic colour scale. The structure of the spectra (single *K*-hole series, double *K*-hole series and resonances) is similar to that of Mg described in Fig. 1. The *K*_α_ spectra for Si, when the LCLS pumps either a pure silicon sample (**d**), a silica sample (**e**) or a mica sample (**f**) are also plotted on the same colour scale: in these spectra, only emission for the first few lines in the single-*K*-hole series is observed, as the experimental LCLS photon energies were always below the *K*-edge threshold for the remaining lines. The high-energy line barely visible in **c** corresponds to *K*_α_ emission from Si ions in the mica sample, whereas the line observed around 1,840 eV in **d**–**f** is the Si *K*_β_.

**Figure 3:**
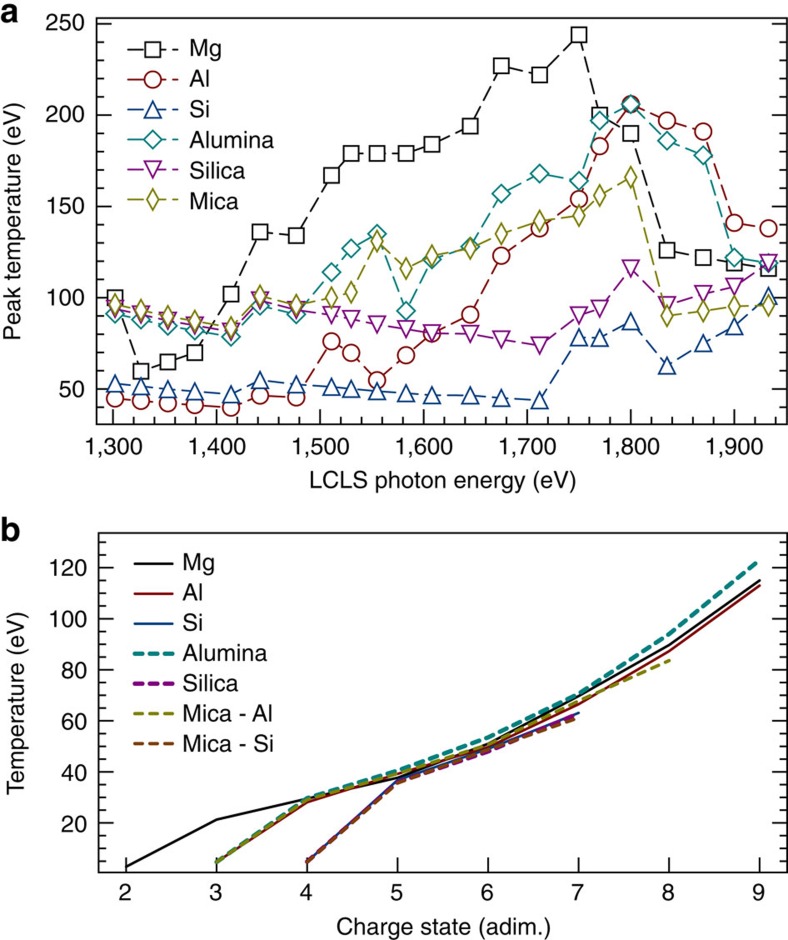
Calculated electron temperatures. The temperatures are determined by time-dependent simulations driven by the focal intensity history of the laser (see Methods section). (**a**) Peak temperatures as a function of the drive photon energies. (**b**) Temperature at the time when the average ionization of the absorbing species (either Al or Si in the mica case) equals the charge state associated with a given line.

The *K*_α_ satellite spectra recorded by a flat crystal spectrometer for Mg are shown in Fig. 1a (the complete data set for all of the different samples can be found in Fig. 2). As the photon energy of the LCLS is progressively increased from the value corresponding to the *K*-edge of the cold material (1,303 eV), the complete series of *K*_α_ satellite lines is observed, including emission from ions with a singly ionized *K*-shell (in the range 1,250–1,360 eV) and from ions with a doubly ionized *K*-shell (1,360–1,480 eV). When the laser photon energy matches the energy of a given *K*_α_ transition, copious resonant emission^22^ is also observed; no resonances were observed at energies corresponding to *K*–*M* transitions, which would indicate a bound *M* shell. Lineouts for the emission of all the lines of the main *K*_α_ series (single *K*-holes) as a function of the laser photon energy are shown in Fig. 1b. As the electron temperature is not high enough to allow thermal ionization of the *K*-shell, the edge features observed in these plots correspond to the measured photo-ionization thresholds for each of the absorbing ions in the plasma. This also means that due to the short lifetime of the core hole states the observed emission from different charge states only occurs during the duration of the FEL pulse. The *K*-edge energies are then compared with those calculated for isolated ions, to extract values for the continuum lowering. For reference, the temperatures for the different targets, calculated by time-dependent simulations of the laser absorption^23^, are shown in Fig. 3.

Unlike the previous results^12^, the contrast between above- and below-edge emission is such that the *K*-edges can be clearly identified for all of the ionic species, in spite of the strong collisional line-mixing effects^11^,^24^ for the highest charge states. As an example, the large increase in emission for the charge state 8^+^ across the *K*-edge in Fig. 1b causes a notable but smaller increase in the emission of the neighbouring charge states, which can be clearly resolved from the larger increases corresponding to their own respective *K*-edges. The same holds for most of the ionic species in the samples containing Al and for ions with an *L*-shell up to three times ionized in the samples containing Si (see Fig. 2); for the latter element, the *K*-edge measurement for higher charge states is limited by the range of photon energies that can be efficiently delivered to the soft X-ray endstation of the LCLS, where the experiment was performed.

### Comparison with analytical models

**Figure 4:**
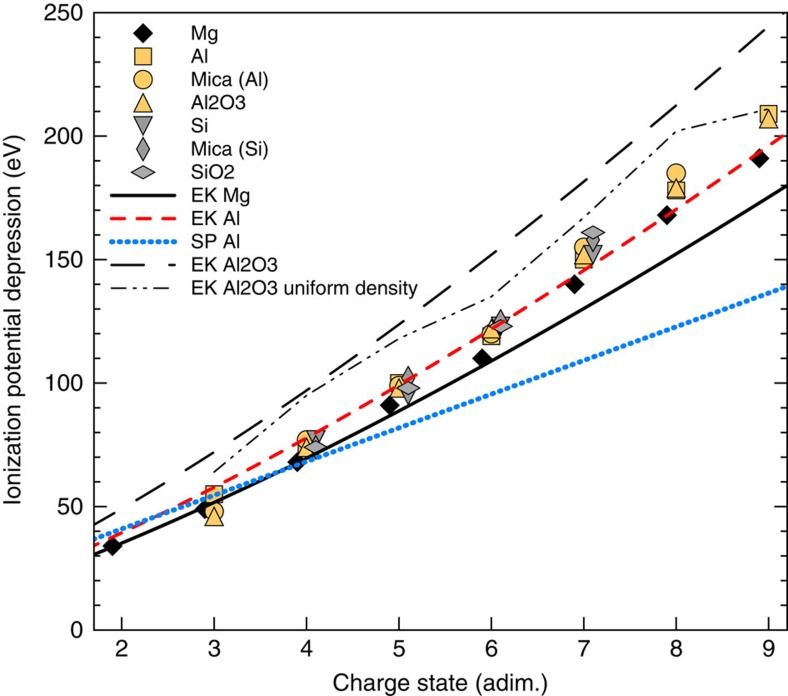
IPD inferred by the *K*-edge measurements. The reduction in the ionization potential of Mg, Al and Si in the different materials is plotted as a function of the ionic charge state and is compared with the predictions of analytical models (EK and SP—see text), assuming a plasma ionization equal to the charge state. The data sets for each different active material are slightly shifted horizontally for clarity. For the alumina, detailed simulations (see Methods section) have also been performed to determine the reduction in the total (uniform) free-electron density of the material due to the different ionization balance of the oxygen ions and the predictions of the EK model in this case are labelled as ‘uniform density'. The experimental resolution in FEL photon energy is 5 eV; thus, the size of the symbols is representative of the uncertainty on the IPD.

The complete set of measurements of ionization potential depression (IPD) for the three elements in different materials is plotted in Fig. 4. The results for Mg and Al are compared with the predictions of the Ecker and Kröll (EK) model (with *C*=1) (refs 1, 12, 25), known to reproduce the previous Al data for charge states 3–7: the new data set confirms the agreement for the lower charge states of these elements, whereas an underestimation of up to ∼15 eV is observed for the highest charge states (the same applies for elemental Si, not shown on the plot). Such discrepancy is larger that the ±5 eV uncertainty due to the accuracy of the measured FEL photon energy. On the other hand, the widely used Stewart and Pyatt (SP) ion-sphere formula^2^ cannot match any of the data sets, which all show a stronger scaling with the charge state of the ion.

A key result shown in Fig. 4 is the insensitivity of the measured IPDs for Al and Si to the different environment in different materials. For fixed average ionization 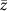 and ion density *n*_*i*_, (mass density divided by the average ionic mass) both the aforementioned EK and SP formulae predict an IPD proportional to the inverse of the Wigner–Seitz radius 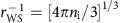. As an example, the ion densities in solid Al and Al_2_O_3_ are 6 × 10^22^ and 1.2 × 10^23^ cm^−3^, respectively, so that a measurement at significantly different densities for the same element is performed. Importantly, in spite of the non-negligible density differences, the three data sets for Al and those for Si almost exactly overlap with each other, in stark contrast with the theoretical predictions. The figure shows the predictions of the EK model for alumina: even taking into account the reduction in the free-electron density (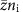) due to the lower atomic number of the oxygen, the model still overestimates the measured IPDs. An experimentalist who should observe a *K*-edge feature in an alumina plasma, and would use this to measure an unknown plasma density by fitting the spectra with the EK model, could then easily introduce an error of a factor of 2 in his/her estimate of the density. Extreme care must then be taken when performing simulations of dense, ionized mixtures, which is of great importance in astrophysics^4^ and inertial-fusion science^20^. Although self-consistent partial ion densities, rather than a single *n*_i_, are typically used in numerical calculations for mixtures^8^, this is generally not the case for plasma codes using analytical continuum-lowering models such as SP or EK.

### Neutral atom approximation

**Figure 5:**
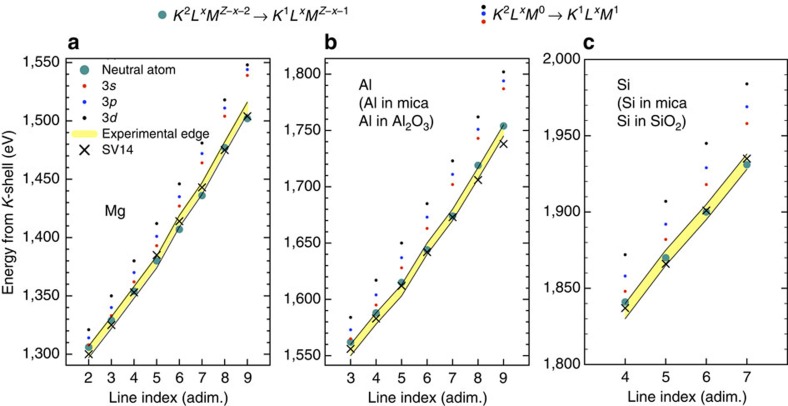
Measured *K*-edges and theoretical predictions. The measured *K*-edges for (**a**) Mg, (**b**) Al and (**c**) Si are compared with *K*- to *M*-shell atomic excitation energies and with the *K*-edges predicted by DFT calculations, using the SV14 approach. The edges for the elements are representative of the full data set (elements and mixtures) and the band represents the experimental uncertainty of ±5 eV, determined as the size of the FEL photon energy binning in the experiment (5 eV, see Methods for further details). The line index equals the charge state of the absorbing ion as defined in the previous figures, that is, if only *K*- and *L*-shell electrons are bound. The measured *K*-edges are found to closely match the *K*–*M* excitation energies in neutral isolated atoms with the relevant number of *L*-shell holes (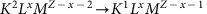). The energies corresponding to the excitation to an initially depopulated *M*-shell (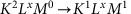) are plotted for comparison.

Further insight can be obtained by directly considering the measured *K*-edges, which are compared in Fig. 5 with atomic excitation energies^26^ and with the values predicted by density functional theory (DFT) calculations, using the method recently developed by Vinko *et al*.^27^ (SV14). The figure shows for clarity only the *K*-edges for the elements, which however agree within the error bars with the *K*-edges for the mixtures. As noted by SV14 in the case of Al, if on the one hand the DFT predictions are in excellent agreement with the data, on the other hand the experimental edges are also surprisingly well reproduced by a much simpler calculation: the energies match the *K*–*M* excitation energies in isolated neutral atoms with the relevant number of *L*-shell holes, where all of the excited electrons, both bound and free, are placed in the lowest energy state in the *M*-shell. We note, out of interest, that this also implies that ions obtained by removing an arbitrary number of *M*-shell electrons from these neutral configurations—the actual ionic configurations to be used in conjunction with an IPD model to describe the hot plasma—have a *K*–*M* excitation energy, which is above the measured ionization threshold; hence, when using any IPD description of the system, the atomic *M*-shell always lies in the continuum and, as a consequence, the continuum in the ionic configurations is lowered by more than the binding energy of any *M*-shell electron (pressure ionization). This also allows for a correct identification of the charge states based on the population of the *K*- and *L*-shell, as used in Fig. 4. In contrast, at much higher temperatures, and therefore weaker coupling, independent experiments^13^ indicate the presence of bound *M* electrons in Al around solid density and it will thus be of great interest to explore the range of applicability of this neutral atom model.

The correspondence between the dense system and free neutral atoms is of course expected for the cold solid-density materials: here, the ion density is fully determined by the atomic properties of the elements composing the crystal and the valence band can be approximated as originating from the overlap of free-atom orbitals from different sites^28^, for both elements and mixtures. Our experimental results provide strong evidence that the same holds even for the hotter, highly ionized samples presented here, which is consistent with the largely temperature-independent valence density of states^27^ predicted by the SV14 calculations. The conduction band for the solid-density plasma thus always forms at the original energy level of the valence shell (*M*-shell) in a neutral Al (or Si), regardless of the neighbouring ions being Al (Si) or O ions, and of the correspondingly different electron and ion densities. From a theoretical point of view, the concept that the electron density that surrounds ions in a dense plasma is similar to that of a neutral pseudo atom has been put forward previously^29^.

## Discussion

Although atomic-like features in the electron density are predicted by both IPD^10^,^27^,^29^ and EOS numerical models^30^,^31^, the physical picture just described is incompatible with models ignoring the overlap of atomic orbitals of neighbouring ions. In particular, this band-like behaviour poses relevant problems for the widespread EOS/IPD models based on a single ion confined in a neutral sphere of radius *r*_WS_ (ion-sphere models)^7^,^8^,^9^,^10^. For the sake of clarity, we note that there is no formal difference between ion-sphere calculations aimed at determining either the IPD or the electronic EOS: the calculation in both cases involves the determination of how the electronic structure of the system varies with density, from which either the position of the continuum (IPD) or the pressure (EOS) is then extracted (an example of the interplay between pressure ionization effects and the compressibility of the Be Hugoniot can be found in the paper by Sterne *et al*.^30^). It has been recently shown^31^ that the chemical potential calculated within the ion-sphere framework can widely differ from that obtained by calculations considering a larger volume of the plasma including neighbouring ions; furthermore, recent self-consistent calculations using the same approximation^10^ exhibit an IPD scaling, which is identical to the SP linear scaling in 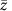, incompatible with the present data. Conversely, the SV14 approach, which is a multicentre model, shows an excellent agreement with the scaling given by the experiment. We thus posit that the usual EOS/IPD models, both analytical and numerical, never benchmarked for highly ionized dense samples due to the lack of experimental data, but still widely used in hydrodynamic and spectral simulations, may yield unphysical results. The data set presented here provides a testbed to benchmark the absorption structure (Fig. 1b) predicted by such models for both elements and mixtures.

## Methods

### Spectrometer

The experimental spectra were acquired on a shot-by-shot basis by using a flat crystal spectrometer coupled to a charge-coupled device detector. We used three spectrometer configurations corresponding to the different spectral windows for the three active elements: the spectra for Mg are acquired with a Beryl (10
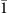
0) crystal, whereas those for Al and Si use the same ADP (101) crystal. The spectra are calibrated using calculated values for a few satellite lines: although the accuracy of the calibration is not crucial for the present analysis, it can be verified by the overlap of the resonant emission with the *x*=*y* line shown on the plots. The spectral resolution is better than 0.4 eV for all the configurations.

### Samples

The thickness of the foils was 1 μm for Mg, Al and Si, 2 μm for silica, 0.5 μm for alumina and 12 μm for the mica targets; the Mg targets were coated by 40 nm of Si on each side, to avoid oxidation. Save for the mica, the thickness is of the order of one attenuation length at the cold *K*-edge of the active element. A fresh portion of the target was irradiated at 45° for every shot, with the spectrometer looking in the normal direction to the surface of the foil.

### FEL photon energies

The nominal laser photon energy was varied in 10 eV steps (15 eV in the case of Mg) and a set of 30 shots was acquired for each material at a given wavelength configuration. The photon energy is inferred for every shot, within a 5-eV error, from the measured LCLS parameters; the energy jitter is found to be such that a few shots are acquired in any 5 eV bin over the whole spectral range. The shots in each of these bins are then summed to get the final spectra, to increase the signal-to-noise ratio, which varies according to the number of shots in each bin.

### Modelling

The laser absorption was modelled by using the SCFLY code^23^,^32^,^33^ to determine the electron temperatures shown in Fig. 3 and the ‘uniform density' IPD values for alumina shown in Fig. 4. The ions are not expected to move or be heated significantly during the LCLS pulse (all samples except the alumina and silica were crystalline). In any case, the temperature does not affect the IPD at high densities^1^,^2^. The spot size of the focused X-ray beam was determined by the offline characterization of ablation imprints^34^ on PbI_2_ samples, whereas the pulse energy is measured on a shot-by-shot basis^35^. The value of the intensity in the focal spot was used in 0D simulations, which use an escape factor formalism for the opacity, and the thickness of the targets as a size parameter; for the mica targets, just two attenuation lengths were used as the thickness in the simulations, to avoid averaging over the colder back part of the thick target, which is not visible in the data. The code was modified to solve the time-dependent rate equations for different elements in the same material at the same time, so that the self-consistent densities and temperatures in the mixtures result from the atomic processes involving all the species in the plasma. The IPD used for the temperature calculations is that estimated by the neutral atom calculations, as described in the study. The SV14 calculations are as described in the relevant paper^27^.

### Data availability

The data that support the findings of this study are available from the corresponding author upon request.

## Additional information

**How to cite this article:** Ciricosta, O. *et al*. Measurements of continuum lowering in solid-density plasmas created from elements and compounds. *Nat. Commun.* 7:11713 doi: 10.1038/ncomms11713 (2016).
